# Firing properties of muscle spindle afferents in the intrinsic foot muscles and tactile afferents from the sole of the foot during upright stance

**DOI:** 10.1113/EP092348

**Published:** 2025-04-10

**Authors:** Thomas P. Knellwolf, Alex Burton, Elie Hamman, Vaughan G. Macefield

**Affiliations:** ^1^ Department of Integrative Physiology, School of Medicine Western Sydney University Sydney New South Wales Australia; ^2^ Human Autonomic Neurophysiology Lab Neuroscience Research Institute Sydney New South Wales Australia; ^3^ Department of Neuroscience Monash University Melbourne Victoria Australia

**Keywords:** cutaneous afferent, foot, free standing, muscle spindle afferent, posture

## Abstract

We review our approach for undertaking microelectrode recordings from the posterior tibial nerve at the ankle, which has allowed us to identify, for the first time, the firing properties of muscle spindle endings in the intrinsic muscles of the foot and of cutaneous mechanoreceptors in the sole during unsupported standing. The responsiveness of muscle spindles in the short muscles of the foot to stretch and related joint movements was similar to that of spindles located in the intrinsic muscles of the hand. Only 27% were spontaneously active in the unloaded condition, whereas 50% were active during unsupported free standing. Moreover, in the latter condition firing rates of 67% of the endings were correlated with changes of the centre of pressure (CoP), primarily (88%) along the anteroposterior axis. The firing of cutaneous afferents supplying the sole of the foot in unsupported free standing depended on the receptor type and location of the receptive field: fast‐adapting type I and slowly adapting type I afferents responded transiently during contact with the substrate on standing and to spontaneous postural adjustments, whereas the tonic firing of slowly adapting type II endings encoded fluctuations in the CoP. We conclude that muscle spindle endings in the intrinsic muscles of the foot are recruited or increase their spontaneous discharge on standing and can faithfully encode changes in CoP during spontaneous or evoked postural sway, a function shared by slowly adapting type II afferents in the sole. These data emphasize the important contributions of sensory sources in the foot to maintaining and responding to perturbations in upright posture.

## Abstract (highlights)

- **What is the central question of this study?**
  We review our work on microelectrode recordings from muscle spindle afferents in the intrinsic muscles of the human foot and of tactile afferents in the sole.
- **What is the main finding and its importance?**
  Most spindle afferents were silent when the foot was unloaded but fired tonically during standing, their discharge covarying with centre of pressure. Most cutaneous afferents responded only during contact and incidental adjustments in posture. We conclude that spindle endings in the muscles of the foot, in addition to tactile afferents from the sole, provide useful proprioceptive information during standing.

## 1 INTRODUCTION

Our vestibular apparatus [the utricle and saccule (otoliths) and semicircular canals] detects changes in head position, but we also require input from the somatosensory system for head position relative to the neck and body to be determined (Lackner & DiZio, 2005). Inputs from low‐threshold mechanoreceptors in muscle and skin provide proprioceptive information to the CNS on muscle length and tension and on skin stretch about a joint; signals from joint receptors can also contribute (Macefield, 2005, 2021; Macefield & Knellwolf, 2018; Proske & Gandevia, 2009, 2012). We do know that the brain has rapid access to sensory information from muscle and cutaneous afferents in the foot (Macefield et al. 1989); therefore, it is likely that these inputs contribute importantly to our ability to stand upright on our feet. We also know that postural sway increases in participants with eyes closed following occlusion of blood flow to the foot, which blocks transmission of sensory signals from muscle and cutaneous afferents (Fitzpatrick et al., 1994; Mauritz & Dietz, 1980). Moreover, selective anaesthesia of cutaneous afferents of the sole of the foot increases postural sway by only ∼11% (Meyer et al., 2004), whereas increases of ∼40%–60% occur in diabetic neuropathy, in which both muscle and cutaneous afferents are affected (Boucher et al., 1995; Simoneau et al., 1994). Accordingly, it is likely that muscle afferents from the foot contribute more to the control of postural sway than do cutaneous afferents, but in the absence of direct data this is speculation.

### 1.1 Roles for muscle afferents in free standing

All muscle spindles in humans are dynamically sensitive to imposed stretch of the parent muscle, usually produced by rotation about the joint on which the muscle acts (Bewick & Banks, 2015; Macefield & Knellwolf, 2018). Muscle spindles faithfully encode joint angle when the muscles are relaxed, there being a linear relationship between joint angle and firing rate (Cordo et al., 2002; Day et al., 2017; Kakuda, 2000; Peters et al., 2017; Vallbo, 1974b) and between joint angle velocity and firing rate (Grill & Hallett, 1995). However, there are no such relationships when the parent muscles are actively holding a joint position (Hulliger et al., 1982, 1985; Vallbo et al., 1981). Recently, ultrasound was used to monitor changes in muscle fascicle length of the tibialis anterior muscle (the long muscle that dorsiflexes the ankle) during slow passive sinusoidal rotations of the ankle joint in humans (Day et al., 2017). The firing of muscle spindles in this muscle was highly correlated with changes in muscle fascicle length, with increases in firing occurring at relatively small, physiological changes in muscle length that were independent of changes in length of the muscle–tendon unit. Given that the tibialis anterior muscle is essentially quiescent during standing, muscle spindles in this muscle would serve as ideal mechanoreceptors for providing sensory feedback on muscle length (Day et al., 2017). Muscle spindles in the calf muscles, which are actively engaged in keeping us upright when standing without support, have also been shown to be sensitive to the types of low‐frequency, low‐amplitude angular excursions associated with standing (Peters et al., 2017).

Although microelectrode recordings from muscle spindle afferents originating in the pretibial flexors have been made from the common peroneal nerve in freely standing humans (Burke & Eklund, 1977), and we know that stretch reflexes in the extrinsic muscles of the foot are essential for maintaining ankle stiffness (Fitzpatrick et al., 1992), these data alone cannot provide a complete picture of the somatosensory neural substrates required for maintenance of the upright posture. Indeed, until now it was not known how muscle spindles in the intrinsic muscles of the foot, which are short muscles with short or absent tendons, behave when the foot is loaded during free standing. We present data on our new recordings from these spindle afferents below.

### 1.2 Roles for cutaneous afferents in free standing

There is a wealth of evidence supporting the role of cutaneous afferents in free‐standing balance and in locomotion. Vibration of the sole of foot has been used to trigger predictable directional body tilts (Kavounoudias et al., 1998). Those authors claim that using a frequency of 100 Hz would selectively activate the cutaneous afferents, based on the understanding that human muscle spindle primary endings primarily respond in a phase‐locked fashion at 80 Hz, but this is not true; we know that human muscle spindles can follow higher frequencies applied directly to the tendon of the parent muscle, as can Golgi tendon organ afferents when the muscles are weakly contracting (Fallon & Macefield, 2007).

To support the relationship between postural control and cutaneous afferent feedback, many studies exist in which this sense is impaired by experimental protocol, age or disease. Immersing the foot in ice to reduce plantar sensation leads to significant changes in gait patterns, affecting joints of the ankle, knee and hip (Eils et al., 2004). Anaesthesia of the metatarsal heads of both feet via iontophoretic application of lignocaine results in predominately mediolateral balance deficits, whereas anaesthesia of the whole foot causes anteroposterior deficits (Meyer et al., 2004). However, in both cases this occurs only when vision is occluded. Decreasing cutaneous reflexes and increasing plantar detection thresholds have been shown to be correlated with age, and this is associated with increased postural sway (Peters et al., 2016). In addition, chronic disease that results in reduced sensation from the foot, such as diabetic neuropathy and Charcot–Marie–Tooth disease, affects standing balance (Kars et al., 2009). Diabetic neuropathy has been shown to affect balance in free‐standing conditions, increasing the area of the centre of pressure (CoP), velocity of CoP and CoP trace length (Uccioli et al., 1995). Both type 2 and severe type 1a variants of Charcot–Marie–Tooth disease have been shown to increase sway area in comparison to healthy control subjects (Nardone et al., 2006, 2000).

Unitary recordings from cutaneous afferents of the sole of the foot have previously been obtained from the tibial nerve via a tungsten microelectrode inserted into the popliteal fossa (Fallon et al., 2005; Kennedy & Inglis, 2002; Lowrey et al., 2013; Strzalkowski, Mildren et al., 2015; Strzalkowski, Triano et al., 2015). Strzalkowski et al. (2018) combined these data and other unpublished data to provide an overview of 401 cutaneous afferents. Low‐threshold mechanoreceptors with small receptive fields (type I afferents; the fast‐adapting FA I and slowly adapting SA I units) were found to have significantly higher unit density in the toes when compared with the arch and heel, and in the lateral arch and metatarsals compared with the medial counterpart. The authors posit that the significance of these regions as the limits of the base of support suggests that these type I afferents play a unique role in maintaining upright posture (Strzalkowski et al., 2018). An earlier microneurographic study of the sural nerve by Trullson (2001) characterized 104 cutaneous afferents, determining unit type and receptive field but finding no significant difference between units of the same class on glabrous and non‐glabrous skin on the lateral aspect of the foot. Importantly, all previous recordings were made in prone participants, which allows access to both the tibial nerve in the popliteal fossa and the sural nerve. Although this positioning is ideal for identifying receptive fields and other mechanical properties, owing to the exposure of the sole of the foot, it is not suitable for assessing the physiological activity of these units in a functional role, such as during free standing. Thus, we developed the means of recording from the posterior tibial nerve so as to allow stable recordings of muscle spindle endings in the intrinsic muscles of the foot, and of cutaneous afferents from the sole of the foot, to be made while participants are standing.

### 1.3 Microelectrode recordings from the posterior tibial nerve

Most unitary recordings during passive and active movements are obtained from peripheral nerves proximal to the site of action, such as the median, ulnar or radial nerves in the upper limb for movements of the hand (Burke et al., 1988; Vallbo, 1971, 1973, 1974a, 1974b), which, given that these nerves are located two joints (wrist and elbow) away, distinct from the supplied muscles, afford great flexibility of the hand to perform behavioural tasks, such as grasping or lifting an object. For the foot, the common peroneal (fibular) nerve is most commonly used, but given that the fibular head is only one joint away (i.e. the ankle), this limits the scope of action; the long (extrinsic) muscles acting on the foot originate from the tibia and fibula, close to the intraneural recording site, hence movement of the microelectrode can occur during movements of the ankle. Moreover, unlike recordings from the median, ulnar or radial nerves in the upper limb, which can target fascicles supplying the skin and intrinsic muscles of the hand, those from the common peroneal nerve can target muscle afferents only from the pretibial flexors and skin on the dorsum of the foot or lateral aspect of the leg. Conversely, the intrinsic muscles of the foot and the skin of the sole are supplied by the tibial nerve, but accessing this nerve in the popliteal fossa means that the knee needs to remain extended at all times; the participant cannot flex the knee to sit down and rest. Recording distal to the ankle joint, from the posterior tibial nerve, avoids some of these issues. Although we have used this approach previously to characterize the firing properties of muscle spindle endings in the intrinsic muscles of the foot (Knellwolf et al., 2018, 2019), we have not done the same for cutaneous afferents supplying the sole of the foot.

## 2 MATERIALS AND METHODS

Data were collected from 10 participants (seven males and three females; 18–30 years of age) over 17 recording sessions. All participants provided written, informed consent. The study was conducted with the approval (#H111010) of the Human Research Ethics Committee, Western Sydney University, with all procedures conducted in accordance with the principles of the *Declaration of Helsinki* (with the exception of registration in a database).

Participants stood on a force plate (Portable Force Platform, AccuGait) that, in turn, was mounted on a motorized platform (custom‐built XY platform, manufactured at Hong Kong University of Science and Technology). The force plate recorded *z*‐axis forces of four quadrants, and *x*‐ and *y*‐axis forces between quadrants. These forces were then integrated to determine CoP changes in the *X* (mediolateral) and *Y* (anteroposterior) directions (Labchart 7, Powerlab 16/35; ADInstruments). The CoP data were then smoothed using a Bartlett window of 2 s. In order for the participants to support themselves, mounted on the platform was a safety bar that rose to chest height. Acceleration of the platform in the *X* and *Y* directions was recorded by a mounted accelerometer. Behind the subject was a bench that allowed participants to sit or lie down if needed. AgCl surface electrodes were attached over the tibialis anterior and soleus muscles to record EMG activity. A goniometer (ADInstruments) was attached to the medial foot and lower leg contralaterally to approximate the ankle angle of the foot from which recordings were being made. An insulated tungsten microelectrode (FHC, Bowdoin, ME, USA) was inserted percutaneously immediately medial to the calcaneal tendon approximately at the coronal plane of the prominence of the medial malleolus, angled anteriorly ∼60° to the normal.

Weak electrical stimuli (0.2 ms, 0.1–1 mA, 1 Hz) were delivered from a constant‐current stimulator (Stimulus Isolator, ADInstruments) through the microelectrode as the tip was advanced manually towards the nerve. A muscle fascicle was identified by the following criteria: (1) electrical stimulation through the microelectrode induced muscle twitches below a threshold of 0.02 mA; (2) passive stretch of the associated digit/s or percussion of the muscle tendon or belly generated reproducible afferent mechanoreceptor impulses; and (3) no tactile afferent response was evoked from light stroking of the skin with a brush. After the fascicle was identified, the microelectrode, if necessary, was adjusted further to isolate a single muscle spindle unit. This was done while passively stretching the digits, allowing identification of non‐spontaneously active units. When evoked by a sustained stretch, muscle spindle afferents demonstrated a characteristic regular firing. Further stretch would increase the mean firing rate, whereas passively unloading the muscle would reduce the mean or abolish the response. After identification of a single muscle spindle afferent, the metatarsophalangeal and interphalangeal joints were passively manipulated in a systematic manner. Initially, a slow circumduction of the metatarsophalangeal (MTP) joint of each digit was implemented, gradually increasing the radius of rotation. If an afferent response resulted from circumduction of a particular digit, this digit underwent further specific manipulations. These were, in order of priority, extension, flexion, abduction, adduction, intorsion and extorsion. Each movement was a ramp‐and‐hold motion to determine minimum and maximum firing rates. Where possible, the proximal interphalangeal joint extension was also tested. Release of the digit was brisk, to assess the presence of an off‐discharge, an increase in firing owing to stretch of the muscle spindle as the muscle returned to its resting length. After these movements, a vibrating probe was applied to the plantar surface or interosseus spaces of the foot over the area of the presumed muscle belly. After a vibration response was tested, the participant was asked to extend and flex their digits weakly, as an isometric contraction against the experimenter's palm. During these tests, the experimenter would also palpate areas of the plantar surface of the foot to check any response to pressure on the muscle belly of the recorded muscle. Where possible, afferents were sorted into primary or secondary endings. Primary units had an irregular spontaneous discharge and/or produced an off‐discharge after sudden cessation of a slow ramping contraction. Secondary units had a more regular discharge rate and did not exhibit an off‐discharge. For non‐spontaneous units, the activity was studied during a sustained stimulus to achieve a constant response.

The criteria used to identify and distinguish cutaneous afferent types were as follows: (1) the participant reported electrical sensations, often described as ‘pins and needles,’ coincident with electrical stimulation through the microelectrode below a current threshold (0.01–0.2 mA) and (2) afferent impulses were produced in response to stroking or palpation of the skin in the innervation territory of the cutaneous fascicle. After recording oligounitary tactile afferent activity, a search was undertaken to identify individual cutaneous mechanoreceptor afferents, defined according to well‐established criteria. As noted above, fast‐adapting type I (FA I) afferents have small receptive fields with well‐defined borders and respond during dynamic phases of contact, generating clear ‘on’ and ‘off’ responses. Slowly adapting type I (SA I) afferents also have small receptive fields with well‐defined borders, responding dynamically to an indentation but also firing during the static phase and, for some afferents, also during the ‘off’ phase. Type II units have larger receptive fields with poorly defined borders: fast‐adapting type I) (FA II) units are highly sensitive, responding to tapping over areas remote from the receptive field, whereas slowly adapting type II (SA II) units respond to lateral skin stretch and have directional sensitivity (Macefield, 1998).

Accessing the receptive field of a cutaneous unit was challenging in the free‐standing participant because receptive fields were on the plantar surface of the foot or toes. To overcome this, after achieving reliable afferent activity, we asked the participant to unload their foot, raising it ∼5 cm above the platform, supporting themselves by holding onto the safety bar in front of them. It was important that the characterization process was restricted to <2 min at a time to avoid fatiguing the contralateral leg. It should be noted that although unloading the participant's foot permitted access to the skin of the foot for unit characterization, it was not possible to test mechanical thresholds with von Frey hairs; moreover, receptive fields were harder to define precisely without a direct line of sight of the sole of the foot. Action potentials were isolated using time–amplitude window discrimination software (Spike Histogram, LabChart 7; ADInstruments).

## 3 RESULTS

### 3.1 Characterization of muscle spindles supplying the intrinsic muscles of the foot in unloaded conditions

The intrinsic muscles of the foot, like those in the hand, are defined by their very small tendons and small actions on a single structure. We know a lot about the behaviour of muscles spindles of the intrinsic muscles of the hand (Burke et al., 1988; Macefield et al., 1990), which respond in only one direction and act about only one joint, responding to angular rotation that leads to stretch of the muscle. Given that the foot has the neural machinery for fine motor control, including rapid sensory and motor transmission to and from the cortex (Macefield et al., 1989), one might expect that muscle spindles in the short muscles acting on the toes would behave in a manner similar to those in the short muscles acting on the fingers. Like muscles in the hand, those in the foot have short tendons, but when flexing the toes they are typically flexed in unison. This is attributable, in part, to mechanical linkage by the superficial and deep transverse metatarsal ligaments, which are attached to the metatarsal heads. Hence, it could be that muscle spindles in the foot are sensitive to remote digit movements.

In addition to muscle spindles acting on the toes, the foot is unusual in having muscles that traverse a large area from the heel to the metatarsals (Tosovic et al., 2012); known as the longitudinal arch, it is deformed during weight bearing and locomotion. These muscles (abductor hallucis, flexor digitorum brevis and quadratus plantae) are likely to play an important proprioceptive role in standing. Indeed, these muscles have been shown to stabilize the foot architecture against gravity (Kelly et al., 2014), and it is reasonable to assume that spindle afferents provide the substrate for detecting the changes in muscle length produced by these forces.

#### 3.1.1 Muscles acting on the big toe

**FIGURE 1:**
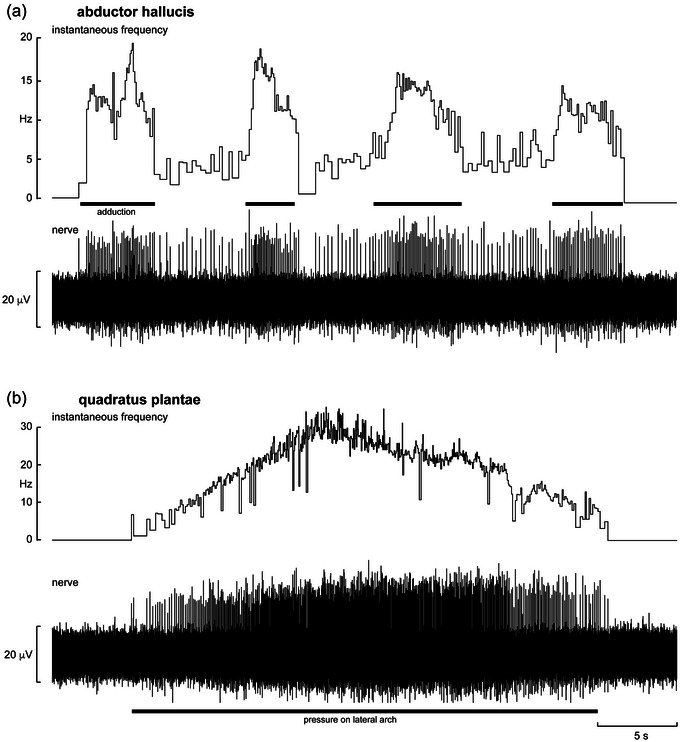
Single‐unit recordings from muscle spindles supplying the abductor hallucis muscle (a) and quadratus plantae muscle (b). Both units were silent at rest and were stimulated by passive manipulations made by the experimenter. (a) The experimenter adducted the first digit, alternating between maximum stretch (indicated by black bars) and stretch at the activation threshold. (b) The experimenter applied a slowly increasing pressure over the belly of the quadratus plantae muscle. When the maximum frequency was achieved, the pressure was slowly decreased and finally removed. Reproduced with permission from Knellwolf et al. (2019).

Unitary recordings were made from three muscle spindle afferents supplying the short muscles acting on the big toe. The spindle illustrated in Figure 1a was located in abductor hallucis; it was silent at rest and was recruited by passive extension, adduction and extorsion movements of the first MTP joint. Another muscle spindle afferent supplying flexor hallucis brevis was spontaneously active at rest, increasing its discharge frequency by extension and abduction of the first MTP joint; slow flexion of the same joint decreased its firing and ultimately silenced the ending. The spindle afferent supplying adductor hallucis was spontaneously active, firing at ∼8 Hz, and responded only to abduction of the first digit MTP joint (i.e., to stretch of the parent muscle).

#### 3.1.2 Flexor digitorum brevis

Recordings were made from three muscle spindle afferents that supplied flexor digitorum brevis; none were active at rest, but they responded to passive extension of the associated MTP joint. A primary ending in this muscle generated an off‐discharge when the second MTP joint was released from flexion, whereas firing rate for another primary ending increased during extension of the interphalangeal joint while the fourth MTP joint was held extended. This ending also generated an off‐discharge after release of abduction and responded to palpation of the plantar surface of the second metatarsal bone.

#### 3.1.3 Lumbricals

Two recordings were made from spindle afferents in the lumbrical muscles, neither of which was spontaneously active. One primary ending, located in the second lumbrical, responded to passive extension, flexion and lateral abduction of the third MTP joint, firing during movement and release of the digit, which caused stretch of the muscle; it also responded to vibration of the plantar surface of the third metatarsal bone. The other ending, located in the third lumbrical, responded strongly to extension of the fourth and weakly to extension of the third MTP and responded to vibration applied directly to the fourth metatarsal.

#### 3.1.4 Plantar interossei

**FIGURE 2:**
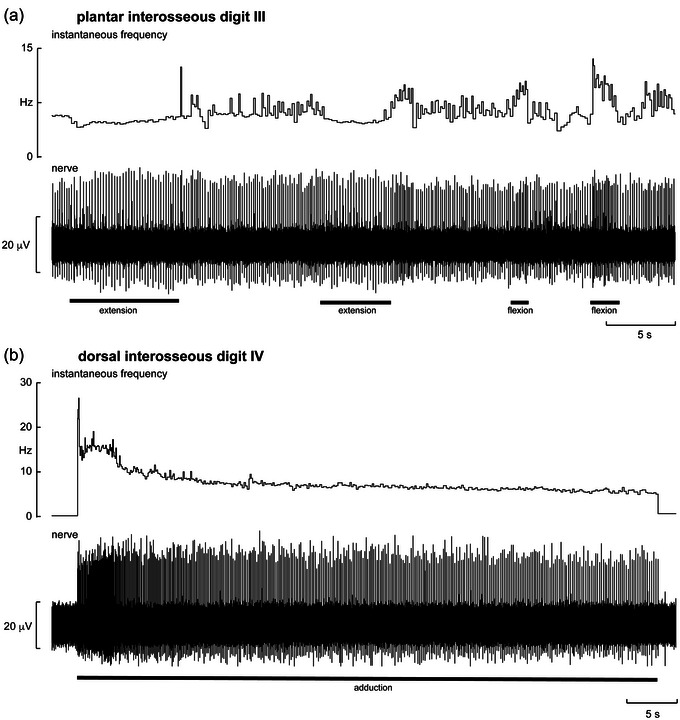
Single‐unit recordings from muscle spindles supplying the third plantar interosseus muscle (a) and the fourth dorsal interosseus muscle (b). (a) The third digit is passively flexed and extended. (b) The fourth digit is passively adducted. The black bars below each trace indicate the period and type of movement. Note that the adduction movement was held at a constant angle throughout the stimulation. Reproduced with permission from Knellwolf et al. (2019).

Four recordings were made from spindle afferents, two of which had a resting discharge, supplying the plantar interosseous muscles. One of the spontaneously active primary endings changed its firing during sustained extension, flexion, medial abduction and lateral abduction of the third toe at the MTP joint (Figure 2a). On release of the stretch there was an off‐discharge that was greatest after medial abduction and weakest after lateral abduction; this ending also responded to vibration applied to the plantar surface of the third metatarsal. Another ending, located in the second plantar interosseous, responded only to abduction (and weakly to extension) of the fourth MTP joint and to vibration applied to the plantar surface between the third and fourth metatarsals, and one ending located in the third plantar interosseous responded to abduction and extension of the fifth MTP joint and weakly to extension of the fourth MTP joint.

#### 3.1.5 Dorsal interossei

There were two recordings made from spindle afferents supplying dorsal interosseous muscles, neither of which was spontaneously active. One, supplying the fourth dorsal interosseous, responded strongly to adduction and weakly to extension of digit IV (Figure 2b). The other, located in the first dorsal interosseous, responded to abduction of the hallux and second toe and to palpation in the dorsal interosseous space between the first and second toes.

#### 3.1.6 Quadratus plantae

Recordings were made from three spindle afferents supplying the quadratus plantae muscle, one of which was spontaneously active at rest. All responded to palpation of the posterior lateral arch anterior to the heel and did not respond to passive movements of any of the toes. Figure 1b shows one of the silent endings responding to slowly increasing and decreasing firm pressure over the muscle belly; another fired erratically and was silenced by palpation, presumably because of its orientation within the muscle belly, and fired several brief bursts as the muscle returned to its resting position.

#### 3.1.7 Flexor digiti minimi brevis

Four recordings were obtained from spindle afferents supplying the flexor digiti minimi brevis muscle, none of which was active at rest. They all responded to extension of the little toe at the MTP joint, and three generated an off‐discharge after release of passive flexion. Another ending responded strongly to vibration on the plantar surface of the fifth metatarsal and weakly to adduction of the fifth MTP joint.

#### 3.1.8 Abductor digiti minimi

Three muscle spindle endings were located in abductor digiti minimi. One was tonically active at rest, increased its firing during extension of the fifth MTP joint and palpation of the posterior lateral arch and was silenced by abduction but produced an off‐discharge when the digit returned to the resting position. The other two endings were silent at rest but responded to adduction (stretch) of the fifth MTP joint and pressure on the lateral aspect of the fifth metatarsal bone.

#### 3.1.9 Unidentified afferents

Two muscle afferent recordings could not be identified, because they had no reliable response to toe movements or palpation, perhaps because sufficient muscle stretch could not be provided. They were, however, both spontaneously active, providing further evidence for ongoing activity of muscle spindles in the intrinsic muscles of the foot in the unloaded condition.

### 3.2 Behaviour of muscle spindles supplying the intrinsic muscles of the foot in free‐standing conditions

Stable recordings from 12 muscle spindle afferents were obtained during unsupported standing, and six were spontaneously active. Two endings were identified initially as the participant sat with the leg pendant and the foot unloaded, but the majority of the recordings were obtained during standing. Of the two endings recorded before participants stood on the force plate, one, located in flexor hallucis brevis, was silent and the other, located in flexor digitorum brevis, had a tonic discharge. We do not have enough data to draw conclusions about how muscle spindles respond to loading of the foot during standing, but in the one recording obtained in the seated position and followed as the participant stood, the spindle was recruited as the weight on the foot increased. It is likely that for those muscle spindles that are spontaneously active in the unloaded condition we would see modulation of their firing rate during the transition from seated to standing, owing to the associated foot deformation.

Of the 10 spindle afferents found while the participant was standing, four were located in flexor digitorum brevis, two in flexor digiti minimi brevis, one in flexor hallucis and another in adductor brevis hallucis. The identity of the muscle for two muscle spindle endings could not be determined.

**FIGURE 3:**
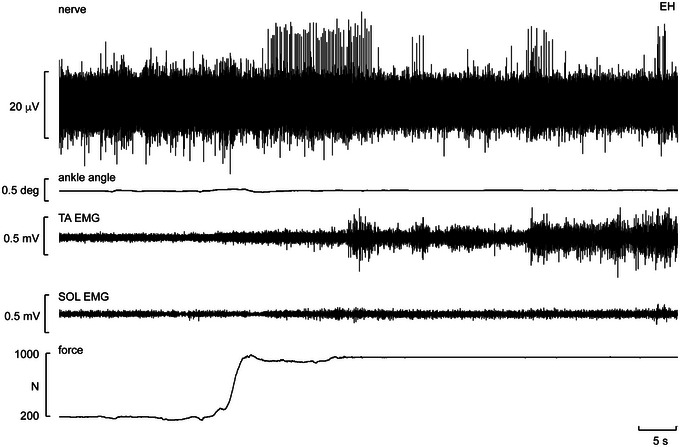
Unitary recording from a muscle spindle supplying the flexor hallucis brevis muscle. The ending was silent at rest and is shown during the transition from the loaded (foot resting on force plate) to the standing condition. Abbreviations: SOL, soleus; TA, tibialis anterior. Reproduced with permission from Knellwolf et al. (2018).

Figure 3 shows a recording from muscle spindle ending located in the flexor hallucis brevis muscle. The afferent was silent at rest, that is, in the unloaded condition, and when the participant was resting his foot on the force plate, that is, in the loaded condition (see baseline force in Figure 3). However, it is apparent that the spindle was not recruited during the transition from seated to standing but while the participant was adjusting his stance. Indeed, it would appear that the firing of this spindle ending was related to the postural adjustments required for standing, indicated by the EMG recorded over tibialis anterior and soleus; this spindle did not generate ongoing activity during standing, which would be expected if it was responding to the high forces applied to the foot and hence strains in the receptor‐bearing muscle. It can also be seen from the goniometer trace that there were very small changes in ankle angle during the transition from the loaded to standing position, emphasizing how stable recordings from the posterior tibial nerve can be.

**FIGURE 4:**
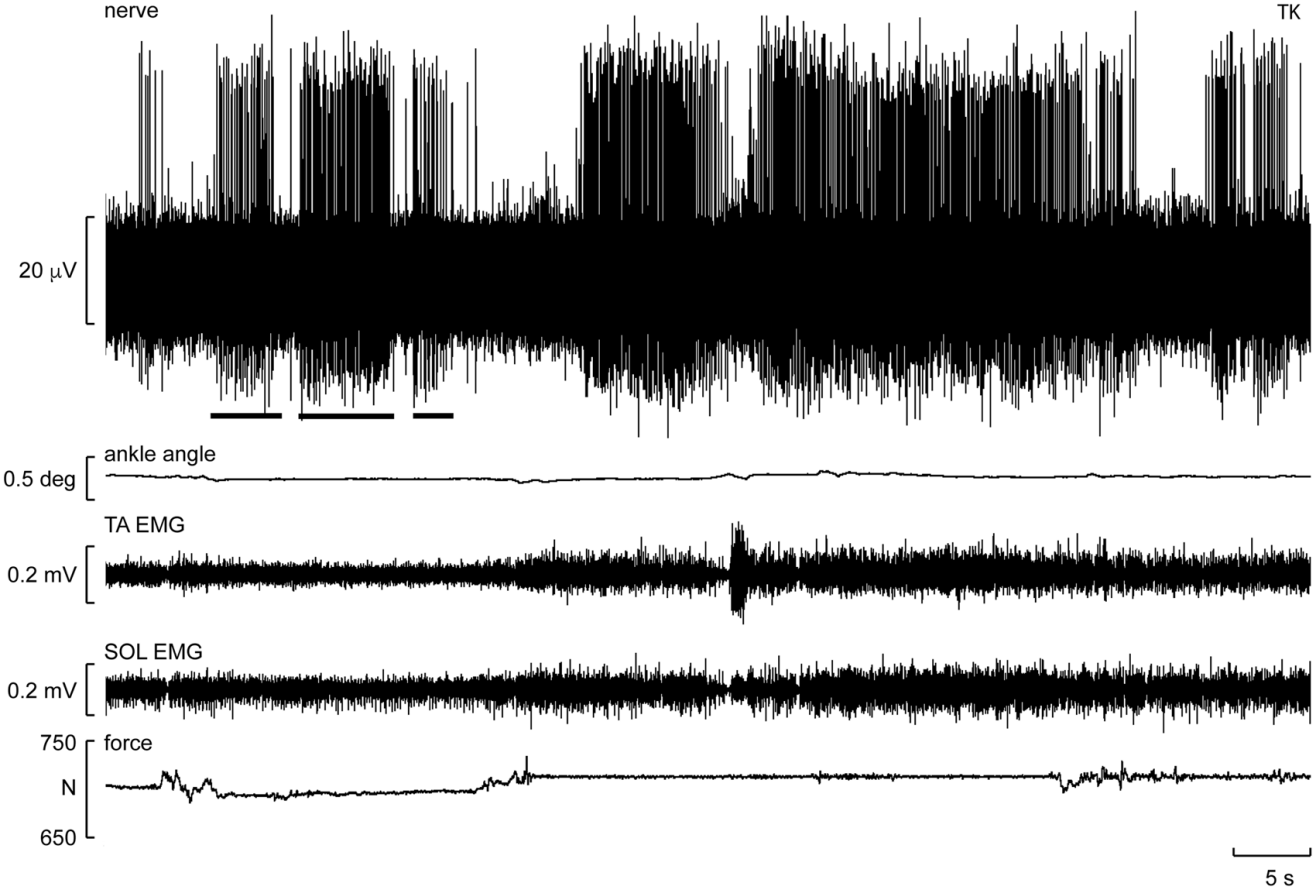
Single‐unit recording from a muscle spindle supplying the flexor hallucis brevis muscle during free standing. Three horizontal bars indicate periods in which the subject was asked to perform weak plantar flexions of the big toe. Abbreviations: SOL, soleus; TA, tibialis anterior. Reproduced with permission from Knellwolf et al. (2018).

Another recording from a spindle ending located in the same muscle is shown from a different participant in Figure 4. This ending was found while the participant was standing, was silent at rest and could be activated by weak voluntary active plantarflexion of the big toe. Spontaneous activation of this spindle appears to be attributable to slight postural adjustments (note the slight changes in ankle angle) that lead to activation of the muscle as a compensatory mechanism. It can also be seen that in the free‐standing condition, as indicated by the increase in EMG of the tibialis anterior and soleus muscles, this spindle ending increases its activity. Although EMG activity could not be recorded from this muscle or from any deep intrinsic muscle of the foot, it is likely that there was also an increase in EMG of this muscle, hence an increase in fusimotor drive that might be attributable to compensatory reactions during postural sway.

**FIGURE 5:**
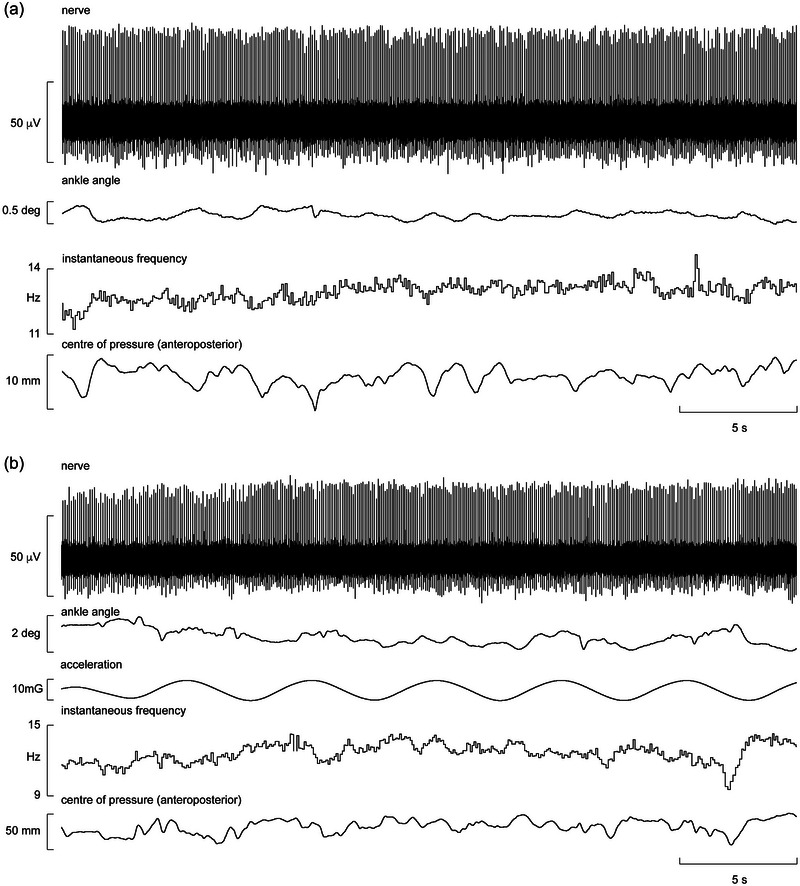
Single‐unit recording from a spontaneously active muscle spindle secondary ending located in flexor digitorum brevis during unsupported free standing. (a) The participant is standing with eyes closed. (b) The platform on which the participant is standing is being moved sinusoidally along the anteroposterior axis. Reproduced with permission from Knellwolf et al. (2019).

Of the 12 muscle spindle afferents recorded during free standing, the discharge of eight covaried with changes in centre of pressure (CoP). Of these, five endings responded to postural sway in the anteroposterior axis, one responded to mediolateral sway and two responded to sway along both axes. An example of a unitary recording obtained from a tonically active secondary muscle spindle afferent located in flexor digitorum brevis during free, unsupported standing with eyes closed is shown in Figure 5a. When the participant was exposed to sinusoidal anteroposterior motion of the supporting platform, indicated by the acceleration signal, there was an increase in the magnitude of sway (Figure 5b). The resultant postural perturbations increased the variation in CoP on the *y*‐axis fivefold, accentuating the frequency response of the spindle ending. And although the maximum modulation of the firing rate of the ending was only ∼5 Hz, this corresponds to ∼45% of the mean frequency of this spindle ending. It is also apparent that there is no overt correlation between spindle firing rate and ankle angle, despite the latter varying with CoP as the body rotates about the ankle.

**FIGURE 6:**
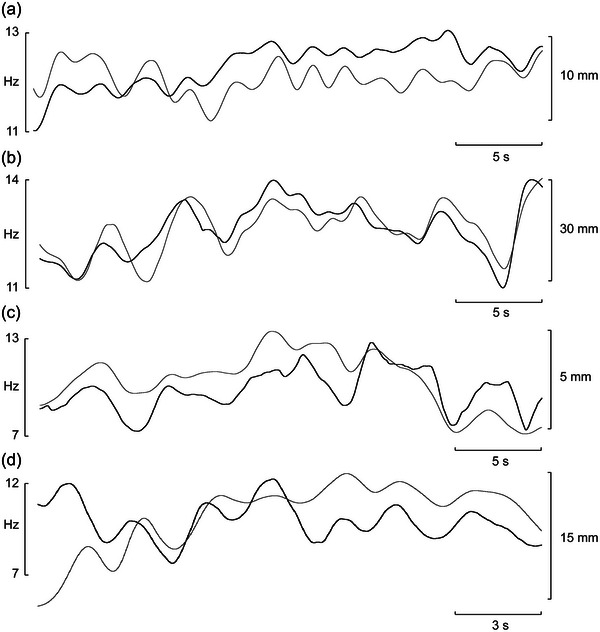
Spontaneous fluctuations in mean frequency of three muscle spindles (thick lines) superimposed onto corresponding changes in the centre of pressure (thin lines) in the anteroposterior plane. The centre of pressure was smoothed using a Bartlett window of 2 s. (a,b) These recordings correspond to the same recordings shown in Figure 5a,b. (c,d) Data from another two units, recorded from flexor digitorum brevis muscle (c) and flexor hallucis brevis muscle (d), are also shown. Reproduced with permission from Knellwolf et al. (2019).

The mean frequency of this muscle spindle afferent is shown superimposed on changes in CoP during free standing with the eyes closed (Figure 6a) and during postural perturbations (Figure 6b), and it is clear that the fluctuation in spindle firing rate covaried with fluctuations in CoP. Mean discharge frequency and the anteroposterior CoP were smoothed using a Bartlett window of 2 s; data from two other spindles are shown in Figure 6c,d. It is apparent that spindle firing rate covaries with changes in CoP, with most turning points and occasionally the magnitude of the traces correlating very well. Moreover, changes in CoP are detected by the muscle spindle over shifts as small as 1 mm.

**FIGURE 7:**
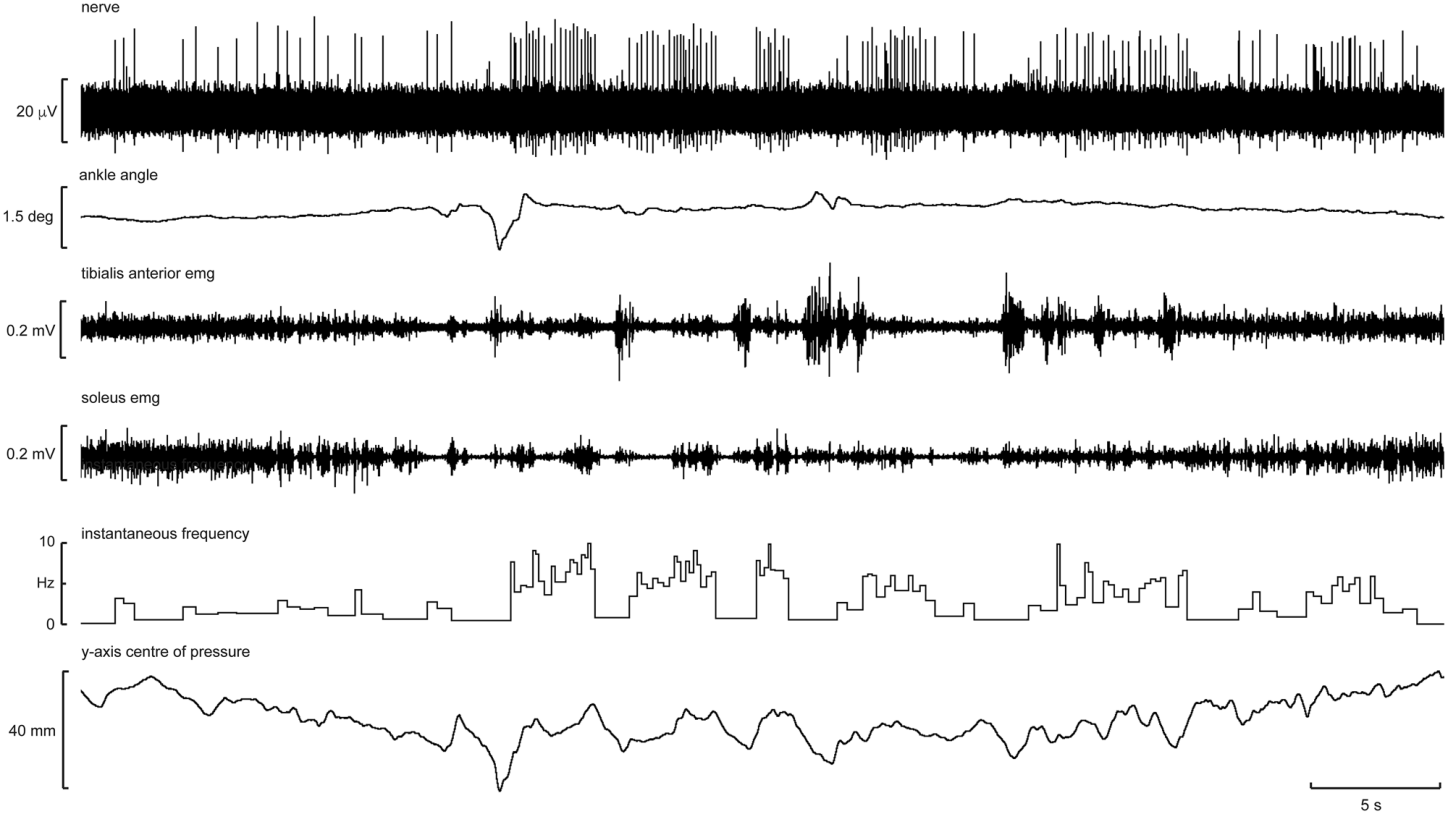
Single‐unit recording from a muscle spindle supplying the flexor hallucis brevis muscle during free standing. Note the covariation of firing with spontaneous fluctuations in the centre of pressure during postural sway. Reproduced with permission from Knellwolf et al. (2019).

Finally, although four muscle spindles were not tonically active during free, unsupported standing, they nevertheless responded to transient changes in posture, apparently encoding the resultant changes in muscle length. An example of a muscle spindle ending located in flexor hallucis brevis that responded to spontaneous adjustments in posture can be seen in Figure 7. Note the very small changes in CoP and ankle angle. We can also see tibialis anterior EMG activity, probably responsible for the anterior CoP shifts, alternating with spindle activation. Given that this pattern begins with a corrective activation to posterior sway, it suggests that spindle activity of the flexor hallucis brevis does not reflect TA muscle activity but rather the resultant muscle stretch associated with the anterior‐directed changes in CoP. In other words, the change in firing is likely to reflect a compensatory contraction of the intrinsic foot muscles for stabilization.

### 3.3 Behaviour of cutaneous afferents from the sole in free‐standing conditions

Based on microelectrode recordings from the tibial and sural nerves in prone participants, the relative densities, distributions, receptive field areas and mechanical thresholds of the four classes of cutaneous mechanoreceptor afferents supplying the glabrous skin on the sole of the foot are well known (Trullson, 2001; Fallon et al., 2005; Strzalkowski et al., 2018). Type I afferents (i.e., those with small receptive fields) innervated by the tibial nerve have a higher density in the toes, lateral arch and metatarsals. Given that these parts of the foot provide the base of support during free standing, one might expect that tactile afferents from these regions of the sole would play a significant role in edge detection, which would add additional information to that provided from muscle spindles in the intrinsic muscles. As noted above, removing cutaneous feedback from the feet has impacts on balance and gait (Meyer et al., 2004; Nurse & Nigg, 1999; Perry et al., 2000). However, as also noted above, any proposed mechanisms by which tactile afferents from the sole of the foot could contribute to the control of upright posture in free standing remain speculative in the absence of data.

#### 3.3.1 Multi‐unit recordings

Because of the difficulty in securing stable recordings from individual cutaneous mechanoreceptors in free‐standing conditions, much of the analysis was limited to multi‐unit recordings, obtained from 28 sites within cutaneous fascicles of the posterior tibial nerve. These recordings were evaluated qualitatively to provide information on the population behaviour of tactile afferents in the sole of the foot, particularly with respect to their capacity to encode loading forces and changes in CoP. As a population, cutaneous afferents in the sole of the foot responded with a large burst of impulses on initial contact of the foot with the force plate. A secondary burst on loading and a burst on unloading were also apparent; in other words, as a population, tactile afferents on the sole responded to the dynamic changes in skin deformation associated with contact, loading and unloading of the foot.

#### 3.3.2 Afferents located on the toes

Fourteen of the multi‐unit recordings had population receptive fields over part of one or more digits; eight on individual digits, six on multiple digits. Only one site presented spontaneous activity. The multi‐unit activity of four sites demonstrated clear correlation with CoP changes in free standing. In all cases, this was during anterior shifts in CoP, and for one recording, activation would occur only at the extreme limits of this movement. This includes the single spontaneous recording that would increase its activity during pressure shifts. There was a latency of ∼10 ms between the nerve signal and the pressure signal, which could represent deformation of the skin occurring prior to pressure applying to the platform. Four of these recordings were not modulated by changes in CoP in free standing. However, they did discharge briefly (∼500 ms) when contact of the receptive field was either made or broken with the platform. This would occur during unsupported free standing as the lateral digits briefly extended spontaneously and when the participant was asked to shift their CoP anteriorly such that the tips of their toes would contact the platform. Four recordings had firing patterns during free standing that were not correlated with either changes in CoP or toe movements. Two recordings were completely silent during free standing and sinusoidal platform movement but responded to passive pressure over their receptive field by the experimenter.

Three recordings had receptive fields located between adjacent digits. None was spontaneously active in free standing. Two recordings, both between digits IV and V, were activated by anterior CoP changes. The third recording, between digits II and III, did not respond to CoP changes, nor to loading or unloading of the foot. However, it was activated during active flexion and extension of the toes, as documented by asking the participant to flex or extend the toes voluntarily. This could potentially represent activation owing to the sheer forces between toes. Finally, one recording had a receptive field over the distal fourth metatarsal. It was spontaneously active during standing and would increase its activity with anterior CoP changes and occasionally with lateral CoP changes. This is likely to reflect increases in pressure over the receptive field.

#### 3.3.3 Afferents located on the medial arch

Four multi‐unit recordings had receptive fields over the medial arch, with some extending to the medial heel. There was an inconsistent pattern of activity, but none was spontaneously active in free standing. Two recordings behaved in a similar manner to those located on the toes, with non‐specific activations that would occasionally be correlated with anterior CoP changes. The receptive field of another multi‐unit site did not contact the platform, and it was silent throughout free standing. Finally, one recording was activated while the contralateral foot was actively adjusted. This could represent a response to deformation of the arch as weight increases over the ipsilateral foot during the shift in posture.

#### 3.3.4 Afferents located on the lateral arch

Four multi‐unit recordings had receptive fields over the lateral arch: two were located anteriorly over the fifth metatarsal and two located posteriorly over the cuboid bone. The posterior recordings did not present spontaneous activity but were both activated by anterior and lateral CoP changes. The receptive field areas of the two anterior recordings were not in contact with the platform and did not exhibit any activity during free standing, with or without imposed platform movement.

#### 3.3.5 Afferents located on the central arch and heel

One recording had a receptive field on the central arch anterior to the heel. It was not spontaneously active but was activated by the posterior extremes of CoP shifts. Finally, one recording was recorded from the calcaneal branch of the posterior tibial nerve and had a receptive field over the lateral heel. It was silent at rest but activated by overt foot movement. This activity was not correlated with any measured changes in CoP.

#### 3.3.6 Unitary recordings

In addition to these multi‐unit recordings, unitary recordings were made from 15 cutaneous afferents supplying the sole of the foot. Afferents were identified according to criteria used to classify tactile afferents in the hand (Johansson & Vallbo, 1979; Vallbo & Johansson, 1984). Five of these were defined as FA I and five as SA I afferents. Five recordings were made of SA II units. No FA II afferents were encountered, no doubt owing to their lower distribution as shown in previous studies. Moreover, given that FA II afferents respond to very brisk events, it is unlikely that they would contribute meaningful information on slow changes in posture. Nevertheless, like those in the hand, we can predict that those in the sole of the foot would respond to contact and release events between the skin and the supporting surface.

#### 3.3.7 Slowly adapting type I units

**FIGURE 8:**
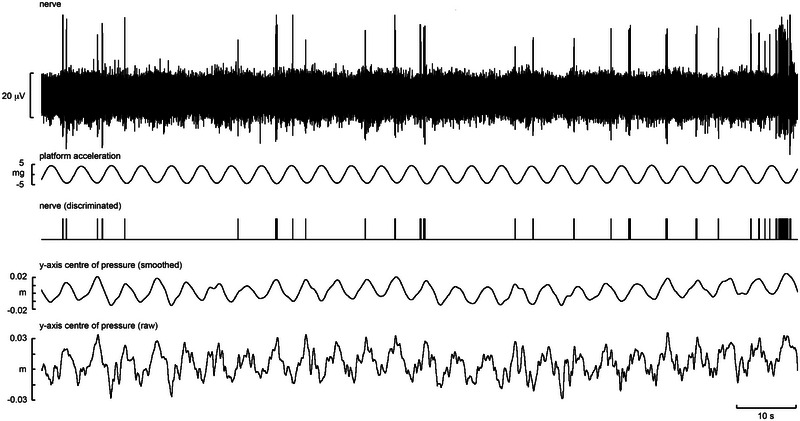
Single‐unit recording from a slowly adapting type I afferent supplying the distal plantar surface of the first digit in a free standing participant during anteroposterior platform movement. Also shown (from top to bottom) is the acceleration of the platform (in mG), discriminated spikes, and smoothed and raw traces of anteroposterior centre of pressure changes (in metres).

Of the five single SA I units recorded, each had varying levels of activity in free standing conditions. All units were on the plantas surface of the digits: two on the third digit, two on the fourth and one on the first. Both units on the third digit were spontaneously active in free standing, firing at a frequency of ∼8 Hz, and could increase to 30 Hz if manipulated passively. However, there was no clear modulation with CoP changes. One unit of the fourth digit would discharge intermittently in a pattern not related to CoP changes, whereas the other was more silent but would clearly modulate with maximal anterior CoP shifts (Figure 8). The unit on the first digit was silent in free standing, with a few intermittent bursts not correlated with measured CoP changes.

Figure 8 displays a recording from a single SA I unit innervating the distal plantar surface of the fourth toe. It was recorded while the participant was in an unsupported, free‐standing position with perturbations applied by a moving platform. By comparing the smoothed trace of CoP changes on the anteroposterior (*y*‐) axis with the platform acceleration, it can be seen that the platform contributed to similar, regular perturbations. The unit's action potentials are clearly correlated with these peaks in negative acceleration and anterior CoP. This demonstrates that the sensitivity to pressure of these units is used in a free‐standing context to indicate movement of the foot in a direction specific to the location of the unit. It should be noted the unit is also seen to fire at other stages of the platform cycle, including once at the extreme positive acceleration. Independent toe movement still providing pressure on the unit, shown by the brief anterior shift in CoP at this point, can explain this.

#### 3.3.8 Slowly adapting type II units

**FIGURE 9:**
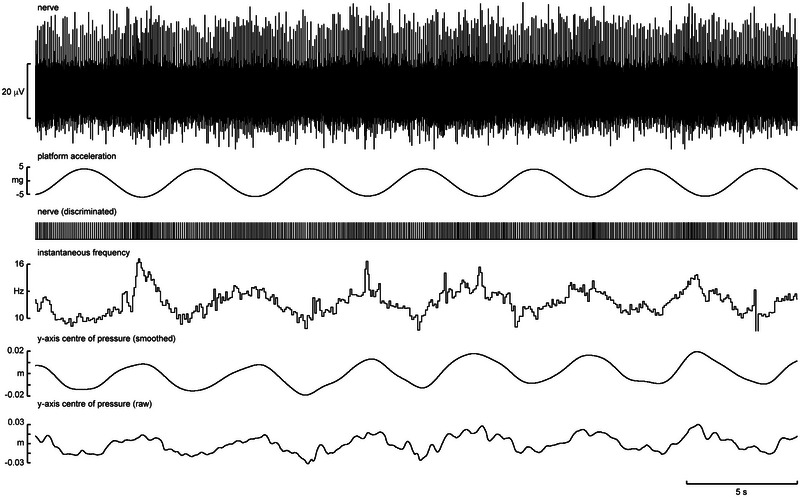
Single‐unit recording from a slowly adapting type II afferent supplying the skin over the second and third metatarsals in free standing with anteroposterior platform movement. Also shown (from top to bottom) is the acceleration of the platform, discriminated spikes, instantaneous frequency of the discriminated spikes and smoothed and raw traces of anteroposterior centre of pressure changes.

The five SA II units recorded also responded variably, but an interesting pattern of behaviour was apparent. Like the muscle spindle endings reported above, spontaneously active SA II afferents demonstrated a clear modulation with changes in CoP during free standing. Figure 9 shows a recording from a single SA II unit supplying the plantar surface overlying the medial three metatarsals. Again, the platform motion can be seen as an effective method of producing predictable shifts in CoP as the body sways with the sinusoidal linear acceleration. By comparing the smoothed trace of CoP with the firing rate of the SA II unit, it is clear that the unit exhibited regular sinusoidal changes in instantaneous frequency between ∼10 and 16 Hz. Given that SA II afferents are known to be sensitive to skin stretch, it might be doing this by encoding the sheer forces on the skin associated with the platform motion. Importantly, changes in afferent frequency were seen to precede CoP changes by ∼100 ms. This could represent signalling the deformation of the skin occurring prior to gross pressure change and is a testament to the sensitivity of these afferents to changes in their mechanical environment. Although further recordings of this phenomenon would be required to support this argument, it can nevertheless be seen that clear and informative single‐unit recordings are possible by recording from the posterior tibial nerve in the free‐standing position, despite considerable subject movement.

Another unit with a receptive field that could not be defined adequately responded in a similar manner with clear anteroposterior CoP changes. One unit on the medial arch showed modulation to mediolateral movement, increasing frequency during lateral CoP changes at a higher mean frequency (∼24 Hz). Another unit with an unknown receptive field fired continuously with no changes with CoP, and one over the fourth or fifth metatarsals showed no spontaneous activity but was activated by pressure and loading of the foot. This spectrum of activity and sensitivity to postural changes suggest that the functional benefit of an individual unit is dependent on the location of its receptive field, the orientation of its receptor and its activation thresholds. Indeed, the powerful encoding seen by some units is likely to be a product of incidental overlap with the CoP parameters measured, while the data encoded from other units might serve a different role.

#### 3.3.9 Fast‐adapting type I units

Three of the five single FA I units recorded were completely silent during standing. In all cases, these units had receptive fields over the lateral or medial edge of different digits that did not contact the platform. Two other units, located on the pad of the fifth digit and the base of the fifth metatarsal, respectively, did activate briefly during free standing, but these activations did not appear to be correlated with CoP on the axes measured. Given that FA I units are sensitive to force velocity, it is expected that there is little activity during zero or constant forces on their receptive fields.

**FIGURE 10:**
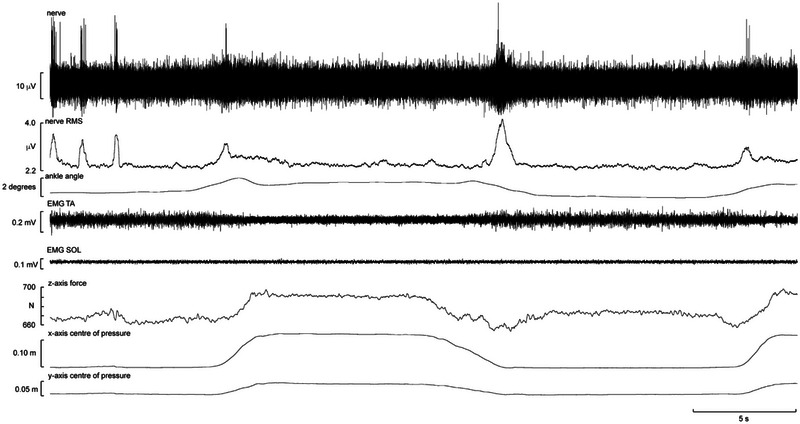
Recording dominated by spikes generated by a fast‐adapting type I afferent responding to pressure on the distal fourth metatarsal recorded in free‐standing conditions, with the ipsilateral foot being slowly loaded, unloaded and loaded again. Also shown (from top to bottom) is the root mean square of the nerve signal, ankle angle, tibialis anterior and soleus EMG, *z*‐axis forces and centre of pressure changes recorded along the *x*‐ and *y*‐axes.

Figure 10 shows a recording dominated by spikes generated by a single FA I afferent with a receptive field over the distal fourth metatarsal, recorded from a free‐standing participant. The activity at the beginning of the recording is the afferent response to the experimenter lightly stroking the receptive field while the foot is unloaded. The participant then loads, unloads and loads the foot again, with the degree of unloading shown by the CoP trace. Afferent responses can be seen at each of these dynamic events. Notably, the afferent response is very brief in comparison to the loading/unloading processes and occurs at the beginning of loading and end of unloading, corresponding to when skin contact is made or broken with the platform.

The number of single‐unit recordings was low, and an examination of the firing characteristics of individual units was beyond the scope of this study. Such an analysis would require recording ≥20 units per unit type over each distribution area across the foot (toes, medial and lateral arches, central arch and heel). However, conclusions can be drawn about the overall pattern of population response from the small sample of single units obtained and the largely qualitative data obtained from multi‐unit recordings: cutaneous afferents in the sole of the foot can encode mechanical transients associated with free standing, and some can encode changes in CoP associated with postural sway. This then leads to the suggestion that tactile afferents from the sole of the foot might well provide useful proprioceptive information that the CNS uses in parallel with inputs from the muscle spindles of the intrinsic foot muscles.

## 4 DISCUSSION

Muscle spindle endings in the intrinsic muscles of the foot behaved in a similar manner to muscle spindles studied in long muscles of the leg and forearm and the intrinsic muscles of the hand which, like those of the foot, have very short tendons (Macefield & Knellwolf, 2018). Having a short or negligible tendon means that in‐series compliance of the muscle–tendon unit is low or absent, potentially allowing a more faithful transfer of mechanical events by stretch receptors in the muscles. Given that we did not attempt to target specific muscles within the foot, the sample sizes within individual muscles were relatively poor, with at most four recordings being made from an individual muscle across participants. This challenge is inherent in the task of attempting to characterize the behaviour of muscle spindles in 10 different muscle groups, which are likely to have distinct and overlapping responses to movement of the toes or conformational changes in the foot associated with loading by the entire body weight. Future studies will need to acquire larger sample sizes within individual muscle groups (∼20 for each), which will be the next step in characterizing the discharge frequencies and variability of these units in free standing and during disturbances to posture.

In the unloaded condition, very few (27%) muscle spindle afferents in the intrinsic muscles of the foot discharged spontaneously, which is similar to the proportion seen in the intrinsic muscles of the hand (Burke et al., 1988) but significantly lower than in the pretibial flexors (Burke et al., 1979). This could represent the differences in incidental resting positions of the joints in these experiments, with ankle plantarflexion in the supine position increasing resting stretch of pretibial flexors in comparison to the resting toe flexion, which would decrease stretch of muscles across these joints. It might also suggest that long muscles of the leg have intrinsically lower thresholds than short muscles of the hands and feet, because the proprioceptive feedback that contributes to postural maintenance is more valuable for muscles acting across the ankle joint than it is for foot stabilization or object manipulation. Moreover, as noted above, the small muscles of the hand and foot, unlike the extrinsic muscles acting on the wrist or ankle joints, or the fingers or toes, have very short tendons; the consequent reduction in in‐series compliance provided by long tendons would mean that there is no slack to be taken up, thereby contributing to their resting discharge properties. With regard to discharge frequency, there was no clear pattern in firing rates across units, hence it is not possible to compare the present data with previous data obtained from extrinsic muscles of the hand (Vallbo, 1974b) or ankle (Macefield et al., 2003). Doing so would require standardization of a resting joint angle for each joint to be monitored with individual goniometers. Differences seen were probably attributable to the difference in resting tension of the muscle.

During unsupported standing, half of the sample of muscle spindles exhibited an ongoing discharge, probably as a result of the increased stretch, owing to loading of the foot exceeding the activation threshold of spindles in the unloaded condition. Importantly, the discharge frequency of each of these spindle endings showed covariation with changes in CoP. Of the remaining spindles that were not tonically active, they were all recruited during postural perturbations, evidently responding to transient changes in muscle length; two of these changes were associated with CoP changes along the recorded axes. These findings suggest that muscles spindles in the intrinsic muscles of the foot form part of the neural substrate for control of upright stance, by way of providing proprioceptive feedback of muscle length and, presumably, the shape of the foot.

Deformation of the arch has been shown to be a function of weight on the knee when seated and of ankle angle in standing (Wright et al., 2012). This suggests that muscles with origins and insertions across the arch, such as abductor hallucis, flexor digitorum brevis and quadratus plantae, would experience greater stretch during standing; muscle spindles within these muscles would be able to report the position of the body over the base of support. The EMG recorded from some of these muscles increases in single‐leg stance compared with double‐leg stance (Kelly et al., 2012) and with loaded weight up to 150% body weight, which is the point at which significant lateral arch deformation plateaus. Additionally, electrical stimulation of these muscles was able to counter the deformation (Kelly et al., 2014). This shows the importance of these muscles in maintaining the shape of the foot, allowing elastic absorption of ground forces.

EMG responses in tibialis anterior and gastrocnemius have been reported during manipulations of the digits and metatarsals, arguing that sensory input from the foot can trigger responses in muscles acting about the ankle (Wright et al., 2012). These results would suggest that muscle spindles in the intrinsic muscles of the foot might be involved in driving these compensatory motor responses. However, the spindles might be playing a simple reactive role to CoP changes instigated by activity in the long extrinsic muscles acting about the ankle joint. At this point, although the relationship of muscle afferent activity and changes in CoP (probably influencing changes in supplied muscle length) is clear, the causal relationship is yet to be determined. As noted above, future studies in which EMG is recorded from individual intrinsic foot muscles would allow us to correlate their discharge with muscle activity and thereby differentiate between changes in spindle firing as a consequence of an increase in fusimotor drive and those that are the consequence of postural adjustments. Of course, it is routine to record EMG from the long muscles from which muscle spindle afferents are being recorded, and this can be done with surface electrodes. For the small intrinsic muscles this is more difficult, requiring as it does invasive intramuscular wires to be inserted into those many small muscles that cannot be accessed via surface electrodes. This was not attempted in the present set of studies because it would add an additional level of complexity to that already faced by developing this new recording methodology of recording from the posterior tibial nerve.

Finally, we know that the sensorimotor control of the small muscles of the foot is similar to that of the hand. Recording somatosensory evoked potentials from the scalp while delivering weak electrical stimuli through a microelectrode inserted into the motor point of abductor hallucis reveals that muscle spindle afferents from the intrinsic muscles of the foot enjoy a rapid sensory transmission to the cortex and a rapid corticospinal motor projection (Macefield et al., 1989).

Although a detailed consideration of the roles of cutaneous afferents from the sole of the foot would require a very large data set, given that there are four types of cutaneous mechanoreceptors in the glabrous skin of the foot, we nevertheless obtained stable recordings in a small number of experiments. Most of these were multi‐unit recordings, which, although they provide limited information content, do give an overall population response of mechanoreceptors in the sole of the foot to loading and unloading and to the effects of postural sway. Although the multi‐unit data were hard to discriminate into individual unit types, a common pattern seen was a mix of FA I and SA I afferents, in which onset and offset of force caused a peak in amplitude from the nerve signal, as seen in FA I units, while the baseline nerve activity increased for the duration of the force as seen with SA I units.

A useful result of the collation of data is elucidating the emerging patterns of activity that occurred in afferent groups with receptive fields in particular regions of the foot. This is understandable, because much of their activity can be predicted from what is known about the afferent responses to punctate stimulation as seen in characterization (Strzalkowski et al., 2018). Unlike muscle spindles found in the long muscles of the pretibial flexors, whose orientation and points of attachment are largely uniform, the characteristic responses of skin afferents to a set stimulus are more variable. This is expected considering the increased complexity of skin deformations experienced during free standing, locomotion and unloaded active movements (Smith et al., 2019). As such, when assessing the function of a receptor to detect the narrow criteria of encoding specific axes of CoP change in relaxed free standing, its response to that stimulus is dependent not only on its intrinsic characteristics but also on its location, depth and orientation in the skin.

With regard to cutaneous afferents innervating the toes, their activity would often occur only during toe movement, either during behavioural movement or as a response to postural change. For example, a rapid posterior change in CoP owing to forward acceleration of the body would lead to a reflex extension of the toes. At points where contact was broken or made with the platform, multi‐unit afferent activity was seen, dominated by dynamically sensitive FA 1 afferents. However, many afferents innervating the toes were either silent during free‐standing conditions or their activity was not correlated with changes in CoP, probably owing to their location.

Perhaps the higher density of cutaneous afferents on the toes (Strzalkowski et al., 2018) facilitates detection of postural disturbances, given that the toes flex and extend in an attempt to stabilize posture by applying pressure to the ground (Tortolero et al., 2008). Minute focal stimulation of the toes has been shown to result in significant changes in balance (Viseux et al., 2018), reinforcing the sensitivity of this area. Despite this evidence, the data collected in the present report suggest that cutaneous mechanoreceptors in the toes are largely not engaged by the task of free, unsupported standing or even during postural disturbance. If they do discharge, it might happen in response to corrective toe movements, rather than informing them. There are two caveats to this interpretation. Firstly, many of the units recorded supplied skin that did not directly contact the ground at rest and would not be expected to be involved in the previously described studies. Secondly, the limited activity seen in a unitary recording is naturally compensated for by the increased density of units in the toes, and they might indeed provide a powerful afferent signal when a population response is considered.

A multi‐unit recording over the ball of the foot responded to both anterior and lateral motion and was spontaneously active during free standing. We have also shown single SA II units being modulated by changes in one axis of CoP. Together, SA I units encoding the constant vertical deformation attributable to body weight and SA II units encoding stretch attributable to sheer forces would provide a neural substrate for both senses of weight and CoP changes. Likewise, units of the medial arch have been seen to respond to both anteroposterior CoP changes and increases in vertical forces as documented during unloading of the contralateral foot. In this case, it is likely that cutaneous stretch detectors (SA II units) would play a greater role than force detectors (SA I units) owing to the reduced contact with the ground and increased deformation of the arch relative to the heel or lateral arch (Smith et al., 2019)

Units located in the lateral arch appeared to subserve a combination of roles. The SA I and SA II units could detect vertical deformation and stretch similar to that seen in the ball of the foot, and FA I units on the lateral foot above the ground would respond when making contact during lateral CoP changes. This indicates that this area would be important for both continuous monitoring of weight and CoP and for edge detection. One unit was recorded from the calcaneal nerve supplying the skin over the sole of the heel. As expected, this unit fired more prominently during posterior motion.

With regard to detecting CoP change, a common trend noted was the change in afferent frequency preceded the stimulating change in CoP often by ∼100 ms. This difference could be a measure of the deformation of the skin preceding the gross adjustment of the foot rather than the skin deforming after the pressure change has taken place. If this is the case, it highlights the sensitivity of these units and usefulness postural maintenance, where rapid detection is vital for a rapid response to destabilization events.

### 4.1 Limitations

Given the complexity of the foot and mechanical constraints, these observations are largely qualitative. For most units there was no measurement of the joint angles; the toes are small and are in close apposition to each other, making measurement of angles difficult. Nevertheless, these studies have the advantage of specifically identifying the supplied muscles using a greater variety of movements. However, without specific angle data, a quantitative analysis of position sensitivity as a function of change (in impulses per second per degree) is not possible, which would provide a valuable comparison with the data previously obtained for the long muscles acting on the hand (Vallbo, 1974b). The superficial and deep transverse metatarsal ligaments are narrow bands that connect the plantar surfaces of the metatarsal heads. Given the mechanical coupling in the foot, it is understandable that some spindles or cutaneous afferents responded to movements of more than one digit, because movement of an adjacent digit could cause deformation of the muscle belly or movement of the associated digit (and overlying skin). For example, one plantar interosseous spindle afferent responded to extension of both the fourth and fifth digits in addition to abduction of the fifth digit. This pattern is understandable with short muscles having their origins and insertions in close proximity. We do not believe that this sample of muscle afferents recorded in the unloaded condition included Golgi tendon organs. However, we know that human tendon organ afferents do not respond to passive muscle stretch when the parent muscle is relaxed but do respond to vibration when the muscle is contracting (Fallon & Macefield, 2007). Given this, it is possible that some of the afferents that were recorded in standing subjects might have been tendon organ afferents, and in the absence of intramuscular EMG data we will not know. Nevertheless, we think it unlikely, given that all presumed muscle spindle afferents, with two exceptions, could be identified by palpation of the muscle belly or stretch of the parent muscle.

It should be noted that, although unloading the subject's foot permitted access to the skin of the foot for unit characterization, there were several limitations. Because of the poor angle, blowing over the receptive field to identify FA II afferents was not possible. Moreover, the restricted time limit for characterization made testing mechanical thresholds with von Frey hairs impractical. Finally, as already commented on, receptive fields were harder to define precisely without a direct line of sight of the sole of the foot.

## 5 CONCLUSION

So, what does this mean? Are cutaneous afferents or muscle spindle afferents more important in postural control? Our observations cannot differentiate between the relative roles of muscle spindles and cutaneous afferents; clearly, both can encode various aspects of upright stance and its perturbation. However, it can be argued that much of the information provided by tactile afferents, with the potential exception of SA II afferents, appears to be incidental (e.g. making and breaking contact with the supporting surface during behavioural or reflex toe movements). It remains to be seen whether stable recordings can be obtained during walking on a treadmill, which is something we are currently embarking on.

## AUTHOR CONTRIBUTIONS

Thomas P. Knellwolf: acquisition, analysis and interpretation of data for the work, drafting the manuscript and revising it critically for important intellectual content; Alex Burton: data acquisition and revising the manuscript critically for important intellectual content; Elie Hamman: data acquisition and revising the manuscript critically for important intellectual content; Vaughan G. Macefield: conception and design of the work, acquisition and interpretation of data, drafting the manuscript and revising it critically for important intellectual content. All authors approved the final version of the manuscript and agree to be accountable for all aspects of the work in ensuring that questions related to the accuracy or integrity of any part of the work are appropriately investigated and resolved. All persons designated as authors qualify for authorship, and all those who qualify for authorship are listed.

## CONFLICT OF INTEREST

None declared.

## Data Availability

The curated data are available on reasonable request.
